# Supplementary material for: Long-term coal gangue dump regulates bacterial communities in different disturbance areas: Evidence mostly from diversity and network complexity
Source: PLoS One. 2025 Oct 14;20(10):e0332127. doi: 10.1371/journal.pone.0332127 (PMC12520416; doi:10.1371/journal.pone.0332127)
Supplement: Attachment — Submitted filename: Response to Reviewers.docx [file pone.0332127.s002.docx]

Dear Editor,

We are grateful to you and the other reviewers for your critical comments and thoughtful suggestions on our manuscript. We have carefully revised the manuscript based on your feedbacks. The revised portions of the manuscript are shown in red. We hope the revised manuscript satisfies the standards of the journal.

Below, we provide point-by-point responses to reviewer#1, reviewer#2 and reviewer#3’s comments and questions. We hope that these revisions are satisfactory and the revised manuscript could meet the publishing standards of the *PLoS One*.

Thank you very much for your assistance with our paper.

1.Language errors should be removed (e.g. "areasf coal" => "areas of coal" (the title of Fig. 6)),

**Response:** Thanks for your suggestion very much, all typographical and grammatical errors have been corrected (e.g., "areas of coal" in Figure 6’s title has been revised to “areas of coal”). The text has been carefully proofread to ensure clarity and accuracy.

2.Presentation problems should be fixed (e.g. quality of Fig. 8, Fig. 9, precision of the title of section 1.4 and figures: 1,5,9; presentation of www links; format of references),

**Response:** Thanks for your suggestion very much, the quality of Fig 8 and Fig 9 has been enhanced for better readability. The title of section 1.4 and the captions of Figures 1, 5, and 9 have been refined for precision. Web links (URLs) have been formatted consistently, and reference formatting has been standardized.

3.Experimental setup should be described in a more detailed way.

**Response:** Thanks for your suggestion very much, a more detailed description of the experimental setup has been described, including methods, equipments, and related references etc. in section of 2.

4.Statistical analysis needs to be presented with more details

**Response:** Thanks for your suggestion very much, additional details on statistical methods, including significance tests and data processing have been included in the section of 2.4(data statistical analysis).

5.Analysis of measurement errors is missing.

**Response:** Thanks for your suggestion very much, we have added note in table 1 and 2(The data in the table are mean±standard error, Different lowercase letters within a column indicate significant differences at the 5% level. Significant difference was performed between that in different disturbed areas of soils.) and in the section of 2.4.

6."Limitations of the study" section would increase the value of the text.

**Response:** Thanks for your suggestion very much, a new section has been added to outline the study’s limitations in section of 5 in line 394-406, providing context for the findings and suggesting directions for future research.

**Responses to Reviewer #1:**

Dear Editor,

We are grateful to you and the other reviewers for your critical comments and thoughtful suggestions on our manuscript. We have carefully revised the manuscript based on your feedbacks. The revised portions of the manuscript are shown in red. We hope the revised manuscript satisfies the standards of the journal.

Below, we provide point-by-point responses to your comments and questions. We hope that these revisions are satisfactory and the revised manuscript could meet the publishing standards of the *PLoS One*.

Thank you very much for your assistance with our paper.

Line 168: All statistical tests used in the analyses should be detailed in the Statistical Analyses section of the Methods, in addition to naming the software. Additionally, the function and purpose of each statistical test should be clearly described. Classification for Nodes role e.g. peripheral nodes, module hubs, connectors and network hubs based on Zi & Pi score are to be presented in the statistical analyses section of the Methods.

**Response:** Thanks for your suggestion very much, we added a detailed description of all statistical tests used, including their functions and purposes. Clarified the classification of node roles (peripheral nodes, module hubs, connectors, network hubs) based on Zi and Pi scores, explicitly stated in the section of 2.4.
 Line 168: Data sorting and analysis. There was no description of data sorting. The title is to be revised.

**Response:** Thanks for your suggestion very much, we revised the section title to better reflect its content (now titled “Data statistical analysis”).

Line 169: The proper citation for the statistical software, including the publisher's name and the specific name of the statistical test for the variance analysis, is to be stated.

**Response:** Thanks for your suggestion very much, we provided full citations for statistical software, including publisher names (e.g., “Version 26.0, SPSS Inc., Chicago, IL, USA”) in section of 2.4.
 Line 171: One or two-tailed test is to be stated.

**Response:** Thanks for your suggestion very much, we revised it in section of 2.4. to “means were declared significant at (p<0.05) according to Tukey’s Honesty Test” in line 171-172.
 Line 173: The URL link is spaced out. To revise to <http://ieg4.rccc.ou.edu/mena/>

**Response:** Thanks for your suggestion very much**,** The URL link has revised to to http:// ieg4.rccc.ou. edu/mena/ in line 181.

Line 175-177: The sentence requires revision. The version of the software is to be stated. Network Analyzer is a built-in plugin/tool within Cytoscape. The links for all the software is to be provided. Origin is useful for statistical analysis and high-quality visualization of bacterial network data; however, it is not designed for network construction or topological parameter computation.

**Response:** Thanks for your suggestion very much, we revised the sentence to accurately describe tools: Stated the version of Cytoscape and clarified that Network Analyzer is a built-in plugin. Removed the incorrect implication about Origin’s role in network construction, added its message Origin (Version9.0, OriginLab Corporation, Canada) in section of 2.4.

Line 207-208 the figures are to be spaced from the phylum name. Subtitle 2.2 – 2.5: to be spaced out from the previous paragraph.

**Response:** Thanks for your suggestion very much, Ensured proper spacing between phylum names and figures. Adjusted spacing between subtitles (2.2-2.5) and preceding paragraphs.
 Line 227: For Shannon Index: Species diversity and evenness.

**Response:** Thanks for your suggestion very much, we revised the Shannon Index is to characterzize species diversity and evenness in line 248.
Line 228: chao1 index (cap C)

**Response:** Thanks for your suggestion very much, Corrected “chao1” to “Chao1” in line 234.
Line 246: Principal Coordinates Analysis (PCoA) is to be stated.

**Response:** Thanks for your suggestion very much, “Principal Coordinates Analysis (PCoA)” has stated in section of 2.4 in line 172-174.

Line 249, Line 272, Line 277, Line 280, Line 289, Line 290, Line 296, Line 298, Line 300: There were some typographical errors e,.g NL,MLS MLZ,MLX , ,while, Pi>0.62),which function.Generally Table 6,these Chlamydiae,Dependentiae and6.42% (to space out)

**Response:** Thanks for your suggestion very much, all typographical errors and formatting have corrected.
 Line 261: MC, (comma to be replaced with dot or word while).

**Response:** Thanks for your suggestion very much, we replaced comma with “while” in Line 261.

Line 268: Two decimal point for R^2=0.8

**Response:** Thanks for your suggestion very much, we added a second decimal to R² values (now “R² = 0.80”) in line 264.
Line 425: MLS. SOC (comma)

**Response:** Thanks for your suggestion very much, we added a comma after “MLS” (now “MLS, SOC”).

Line 302: Discuss

**Response:** Thanks for your suggestion very much, we revised” Discuss” to” Discussion” in line 283.

Figure 4: *, ** and *** to be denoted/described in the figure footnote.

**Response:** Thanks for your suggestion very much, added a legend to denote statistical significance markers (Note: ***represents p < 0.001, ** represents p < 0.01, * represents p < 0.05) of Figure 4.
 Figure 8: Too small, blur and hard to visualize.

**Response:** Thanks for your suggestion very much, we have enlarged the figure and enhanced its resolution to ensure better visibility and eliminate blurriness, and provided their PDF.
 Figure 9: The diagram is a bit small. Axis are to be denoted in the footnote. Network topological properties (metrics table), taxa, modules, validation could be presented apart from Figure 9.

**Response:** Thanks for your suggestion very much, the diagram has been resized for improved readability. Axis notations have been denoted in the footnote. Network topological properties taxa were in table 5.

References did not conform to the journal format.

**Response:** Thanks for your suggestion very much, we have revised the paper’s format.

Reviewer #2:

Dear Editor,

We are grateful to you and the other reviewers for your critical comments and thoughtful suggestions on our manuscript. We have carefully revised the manuscript based on your feedbacks. The revised portions of the manuscript are shown in red. We hope the revised manuscript satisfies the standards of the journal.

Below, we provide point-by-point responses to your comments and questions. We hope that these revisions are satisfactory and the revised manuscript could meet the publishing standards of the *PLoS One*.

Thank you very much for your assistance with our paper.

1- The abstract needs to be rewritten to clarify the results of the work with numerical values that confirm its efficiency.

**Response:** Thanks for your suggestion very much, we have rewritten the abstract to clarify the key results, including specific numerical values that highlight significance of our findings in line 28-57.

2- The introduction is not numbered, and the researcher's contributions are not clearly stated. The introduction also requires a paragraph explaining the structure of the research in all its sections.

**Response:** Thanks for your suggestion very much, the introduction has been restructured with numbered for better clarity in line 60. The researchers’ contributions are now explicitly stated. A new paragraph has been added to outline the structure of the paper in line 107-120.

3- A section detailing a related works of the most important research presented in the same field for a period of time extending between 2020-2025.

**Response:** Thanks for your suggestion very much, we have been added the most relevant and recent literature in the coal gangue field, with a focus on advancements between 2020 and 2025 to the introduction in line 65-89.

4- Discussing the results clearly reflects the researcher's understanding of the work he has presented.

**Response:** Thanks for your suggestion very much, the discussion has been refined to more clearly articulate the implications of our findings, ensuring a deeper interpretation of the results.

Reviewer #3:

Dear Editor,

We are grateful to you and the other reviewers for your critical comments and thoughtful suggestions on our manuscript. We have carefully revised the manuscript based on your feedbacks. The revised portions of the manuscript are shown in red. We hope the revised manuscript satisfies the standards of the journal.

Below, we provide point-by-point responses to your comments and questions. We hope that these revisions are satisfactory and the revised manuscript could meet the publishing standards of the *PLoS One*.

Thank you very much for your assistance with our paper.

1. English Language and Clarity

- While the manuscript is generally understandable, it contains multiple grammatical issues, awkward phrasing, and non-standard expressions.

- For example:
- “we took the coal gangue storage site… as the research object” → “we selected the coal gangue storage site… as the study area.”

- “bacteria and bacteria” → “interactions among bacterial taxa” or “microbial taxa.”
- A professional English language editing service is highly recommended to enhance readability and clarity.

**Response:** Thanks for your suggestion very much, the manuscript has undergone thorough language editing to correct grammatical errors and enhance overall readability by inviting a professional editing service to ensure linguistic precision. Some expression has been revised based on your suggestion. Such as “We took the coal gangue storage site… as the research object” has revised to “We selected the coal gangue storage site… as the study area.” “Bacteria and bacteria” revised to “Interactions among bacterial taxa.”

2. Abstract
 - The abstract is informative but overly detailed. Consider condensing it by summarizing key results more concisely.

- Please clarify statements such as “flora under the phyla with lower abundance may be the key node” for better reader comprehension.

**Response:** Thanks for your suggestion very much, the abstract has been revised for conciseness while retaining key results. Ambiguous statements have been modified its expression for better comprehension. (e.g., “flora under the phyla with lower abundance may be the key node” has been revised to “the bacteria with higher relative abundance may be not the key node in the bacterial molecular ecological network, while bacteria with the lower relative abundance maybe” in line 49-51).

3. Figures and Tables:

- Figures are informative, but some require better labeling and legends for standalone interpretation.

- Ensure all abbreviations (e.g., MC, NC, SOC) are clearly defined when first used in each figure/table and in the main text.

**Response:** Thanks for your suggestion very much, we enlarged for better visibility for clarity and added footnotes to Figures. All abbreviations (e.g., MC, NC, SOC) are now clearly defined upon first use in both figures and text.

4. Discussion:
- The discussion section is descriptive but would benefit from a clearer separation between discussion and results.

- Consider integrating more critical comparisons with related literature to emphasize how your findings advance existing knowledge.

**Response:** Thanks for your suggestion very much, the section has been reorganized to clearly distinguish between results and discussion, additional comparisons with recent literature have been integrated to emphasize how our findings advance current knowledge in section of 4.1-4.4.
 5. Conclusion:
 - The conclusion summarizes the findings well, but a final remark on the potential implications for ecological remediation or future research directions would enhance its impact.

**Response:** Thanks for your suggestion very much, the conclusion now includes our study limitation and future research directions in line 404-416.
 Minor Suggestions:
 - Double-check the formatting of references to ensure consistency.

**Response:** Thanks for your suggestion very much, we have carefully revised the formatting of references to ensure consistency.
 - Clarify whether ethics approval or permits were required, especially for field research.

**Response:** Thanks for your suggestion very much, ethical approval or licensing is not required for the study.
- Include DOIs for all references where available.

**Response:** Thanks for your suggestion very much, we have included DOIs for all references where available except for references of 9, 30, and 54 because they have no DOI.

We hope these revisions have addressed all concerns raised by the reviewers and significantly strengthened the manuscript. Thank you for your valuable time and insightful suggestions.

Sincerely

Dongsheng Jin

Shanxi Agriculture University
