# Supplementary material for: Long-term coal gangue dump regulates bacterial communities in different disturbance areas: Evidence mostly from diversity and network complexity
Source: PLoS One. 2025 Oct 14;20(10):e0332127. doi: 10.1371/journal.pone.0332127 (PMC12520416; doi:10.1371/journal.pone.0332127)
Supplement: Attachment — Submitted filename: Response_to_Reviewers_auresp_2.docx [file pone.0332127.s003.docx]

Dear Editor,

We are grateful to you and the other reviewers for your critical comments and thoughtful suggestions on our manuscript. We have carefully revised the manuscript based on your feedbacks. The revised portions of the manuscript are shown in red. We include the figure legends and tables within the manuscript. We hope the revised manuscript satisfies the standards of the journal.

Below, we provide point-by-point responses to reviewer#1, reviewer#3 and reviewer#4’s comments and questions. We hope that these revisions are satisfactory and the revised manuscript could meet the publishing standards of the *PLoS One*.

Thank you very much for your assistance with our paper.

**Responses to Reviewer #1:**

Spacing/caps required for the following:

Line 139: area(LFA)

**Response:** Thanks for your suggestion very much, we have added spacing and corrected it to area (LFA) in Line 145.

Line 187: Principal Coordinate Analysis

**Response:** Thanks for your suggestion very much, “Principal coordinate analysis” have corrected to “Principal Coordinate Analysis” in line 200.

Line 189: Canoco 5.0, Canoco 5 Support Site [www.canoco5.com](http://www.canoco5.com)

**Response:** Thanks for your suggestion very much, we have added spacing and punctuation [www.canoco5.com](http://www.canoco5.com) in line 203.

Line 197: Origin (Version9.0, Origin Lab Corporation

**Response:** Thanks for your suggestion very much, we have added spacing in line 210.

Line 314: soil[13,14]

**Response:** Thanks for your suggestion very much, we have added spacing and corrected to soil [13,14] in line 381.

Line 315: distance[19,22]

**Response:** Thanks for your suggestion very much, we have added spacing and corrected it in line 382.

Line 312: reference bracket ( ) to be replaced with [ ]

**Response:** Thanks for your suggestion very much, all reference brackets in the manuscript have replaced as required.
Fig to be replaced with Fig. or Figure throughout the manuscript.

**Response:** Thanks for your suggestion very much, “Fig” has been replaced with “Figure” consistently throughout the manuscript.

**Responses to Reviewer #3:**

Language Polishing: A final proofreading pass could further enhance clarity, especially in the abstract and discussion sections where some phrasing remains slightly awkward (e.g., “may be not the key node” → “may not be the key node”).

**Response:** Thanks for your suggestion very much, we have corrected awkward phrasing (e.g., “may be not the key node” → “may not be the key node” in line 18 and line 52) and refined sentence structures for improved readability, conducted a full proofreading pass to ensure consistency, grammatical accuracy, and fluency across the entire manuscript, especially in key sections such as the abstract and discussion.

Figure and Table Legends: Ensure that all abbreviations and statistical markers (e.g., \*, \*\*, \*\*\*) are clearly defined in figure legends for standalone comprehension.

**Response:** Thanks for your suggestion very much, all abbreviations used in figures and tables are now clearly defined in their respective legends to ensure standalone comprehension; symbols for statistical significance are now explicitly explained in each legend (e.g., *represents p < 0.05, ** represents p < 0.01, *** represents p < 0.001”).

Literature Context: The introduction now includes recent studies (2020–2025), which is excellent. A few more comparative insights in the discussion, especially contrasting findings with studies from similar post-mining contexts globally, could strengthen the interpretation further.

**Response:** Thanks for your suggestion very much, according to your suggestion, we have added comparative insights in the discussion (e.g., in line 371-373, 448-457).

**Responses to Reviewer #4:**

1. Language problems:
1.1 there was no significant differences => there were no significant differences

**Response:** Thanks for your suggestion very much, “there was no significant differences” has been corrected to "there were no significant differences" in line 236.

1.2 while bacteria with the lower relative abundance maybe.
=> while bacteria with the lower relative abundance might have been.

**Response:** Thanks for your suggestion very much, “while bacteria with the lower relative abundance maybe” has been corrected to “while bacteria with the lower relative abundance might have been” in line 19 and line 54.

1.3 "From Table 5 and Fig 8, the positive correlation between bacteria in different disturbed areas accounted for more than 58%, indicating that cooperative cooperation was dominant and competition was weak."
=>
"As shown in Table 5 and Figure 8, positive correlations between bacteria in different disturbed areas accounted for more than 58%, indicating that cooperative interactions were dominant, while competitive interactions were weak."

**Response:** Thanks for your suggestion very much, we checked the table, “From Table 5 and Fig 8” has been corrected to “As shown in Table 4 and Figure 8”in line 331.

1.4 "Species play an important role in the process of material circulation, energy flow, environmental pollution indication and purification by forming complex network systems In the natural environment"
In => in

**Response:** Thanks for your suggestion very much, “In the natural environment" has been corrected to “in the natural environment” in line 416.

We hope these revisions have addressed all concerns raised by the reviewers and significantly strengthened the manuscript. Thank you for your valuable time and insightful suggestions.

Sincerely

Dongsheng Jin

Shanxi Agriculture University
